# Covid-19 pandemic effects and responses in the Maasai Mara conservancy

**DOI:** 10.1177/14673584231162275

**Published:** 2023-03-13

**Authors:** Shreya Chakrabarti, Anneli Ekblom

**Affiliations:** Program Associate - Space for Giants African Conservation; Archaeology and Ancient History, Uppsala University, Sweden

**Keywords:** Covid-19, nature tourism, masai-mara conservancy, conservation, community conservation

## Abstract

Local comparisons of effects, responses and mitigations to the Covid-19 pandemic are of vital importance in building a sustainable tourism. This is particularly the case for conservancies in Africa which is largely dependent on international tourism. Qualitative interviews were carried out in the Kenya Maasai Mara Wildlife Conservancies Association (MMWCA)with landowners, lodge managers and staff, tourism operators, community organisations and NGOs between January and May 2021. The MMWCA is an important case study as conservancies pay lease payments to more than 14,528 landowners through tourism revenues. The results show how partner conservancies took different paths in securing payments of leases and salaries by rotating staff, attracting international funding and by targeting domestic tourism. Meanwhile, landowners experimented with alternative economic activities such as cattle herding and diary production. The study shows the strength of MMWCA as a stakeholder partnership to proactively design measures including renegotiation of lease-payments, in soliciting external funding and in re-distributing funding. The positive role of domestic tourism is also stressed. The pandemic brought to the forefront discussions on equity and benefit sharing and on the sustainability of the model itself. Recommendations are given to strengthen possibilities for alternative incomes sources and for a diversification of strategies of the MMWCA partners, including the need to stimulate domestic tourism as a parallel source of income. These recommendations are also relevant to conservation areas across the African continent.

## Introduction

**Figure 1.**
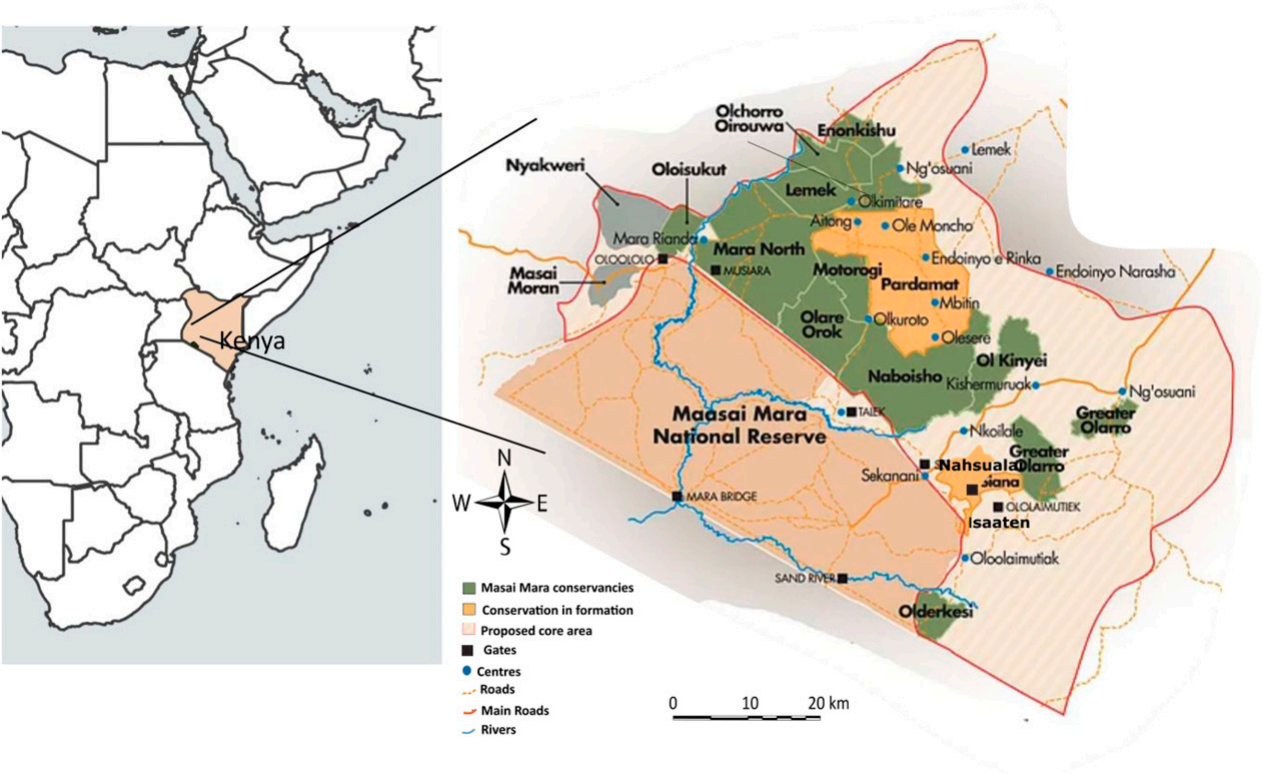
Location of Kenya and the Greater Mara Conservancy, which encompass the national reserve, a number of conservancies and the conservancies which form the Kenya Maasai Mara Wildlife Conservancies Association (MMWCA) (modified from Maa Trust, 2020 which is one of the NGO partners in the Greater Mara Conservancy).

The responses and local strategies to counter the decline in international tourism with the Covid-19 pandemic reveal important lessons for rethinking tourism on several scales (Gössling and Higham, 2020; Gössling et al., 2020; Niewiadomski, 2020; Prayag, 2020). Nature-based tourism and linked hospitality services on the African continent are especially sensitive to global economic and political fluctuations they are typically highly dependent on international and advance bookings (Hockings et al., 2020; Lindsey et al., 2020; Mudzengi et al., 2022). This is especially the case in conservancies such as the Kenya Maasai Mara Wildlife Conservancies Association (MMWCA), an association of independent conservancies which are part of the Greater Mara Conservancy (Figure 1). Here local economies are geared towards tourism and benefit sharing schemes are typically based on community projects funded through revenue. It is therefore of importance to study the effects of the decline in international tourism and rapid loss of revenue. The MMWCA is an especially interesting example as it presents an alternate model of nature-based tourism and community partnerships. Communal lands adjacent to the state-managed Maasai Mara National Reserve (MMNR) are leased from landowners by tourism hospitality operators in return for lease payments. Lease payments currently support more than 14,528 landowners (MMWCA, 2019). About 90% of the conservancies in the Maasai Mara depend solely on international tourism and in 2020 the Covid-19 pandemic resulted in an almost total loss of their revenue. International travel fell by an estimated 22% in the first quarter of 2020 (Kenya News Agency. 2021; Fotiadis, et al., 2021) as whole international tourist arrivals in Kenya declined by −71% compared to previous years with a 46% decline in receipts (USD), a number which kept decreasing in 2021 (UNWTO, 2022). International flights were reinstated on 1 August 2020, with 14-days quarantine for some countries and mandatory testing and new restrictions with second and third waves in November 2020 and April 2021 (Mukami Muragu et al., 2021). As a result of declining revenues, tourism hospitality operators struggled to meet their commitments to the landowners.

The local experiences of these challenges are crucial for an understanding of how to ensure long term economic and social stability for hospitality services and local communities in conservancies funded through revenues. In this paper we analyse the effects of the economic impacts from the 2020 Covid-19 epidemic based on 20 qualitative interviews with tourism operators, lodge managers, lease owners, community organisations and NGOs, carried out between January and May 2021. Here we ask: what strategies for mitigations were taken in the MMWCA, what were the short-term and more durable responses among selected actors, and how did tourism operators and landholders compensate for lack of income? What have been the responses to the economic challenges and the outstanding lease payments, and what are the main lessons learned post-pandemic for MMWCA and other conservancies?

## Litterature review

This paper is related to the emerging field of studies on the Covid-19 pandemic and what we can learn when it comes to a more sustainable tourism (see for instance Aronica et al., 2022; Fotiadis et al., 2021; Niewiadomski, 2020; Prideaux et al., 2020). International tourism is sensitive to global political changes such as economic trends, terrorist attacks and epidemics, however the effects of Covid-19 on decline in international tourism has been higher than ever before measured (Duro et al., 2021; Gössling et al., 2020; UNWTO, 2022). The effects of international trends, including the Covid −19 crises, varies based on the organisation of the tourism market locally and nationally (Duro et al., 2021; Gaffney and Eeckels, 2020; Rogerson and Rogerson, 2020). Local studies are needed that look at specific challenges and also examples of mitigations case by case. Nature based tourism represents a third of all tourism in Africa (Spenceley et al., 2021). Thus, conservation areas in Africa are particularly interesting cases to study as they are highly dependent on international tourism and much of the surrounding economy is also based on tourism making them highly vulnerable to trends in international tourism (see Lindsey et al., 2020; Hockings et al., 2020; Mudzengi et al., 2022).

In a worldwide comparison of effects and responses to the pandemic in conservation areas Spenceley et al. (2021) concluded that although financial losses had severe repercussions on these areas, there are also positive lessons to be learned. Wildlife expanded in areas which otherwise have heavy tourism, some conservation areas expanded virtual tours and communications, incomes were diversified. These are all changes which can help create a more viable nature-based tourism. In addition, domestic tourism did compensate for some of the losses. The importance of domestic tourism in conservation areas on the African continent is under appreciated. Prior to the Covid-19 domestic tourism was on the rise and had compensated for earlier ‘dips’ in international arrivals (Bitok, 2020). In a unique worldwide study of low-income countries prior to the Covid-19 pandemic income redistribution and overall poverty mitigation is suggested to be higher from domestic tourism than international tourism (Llorca-Rodríguez et al., 2020). The Covid-19 pandemic more than ever before stressed the importance of marketing conservation areas also to the domestic public (see for instance Melubo, 2020; Rogerson and Baum 2020; Soliku et al., 2021; Woyo, 2021; Hambira et al., 2022), as will also be discussed here in the context of Masai Mara.

The conservancy model of Kenya Maasai Mara Wildlife Conservancies Association (MMWCA) makes it particularly interesting as a case study as landowners receive direct benefits from tourism hospitality operators, land-lease payments and other benefits such as school fee bursaries and preferential employments in the camps. This study therefore relates to a broader discussion on how conservation would best be organised. The model follows a trend of decentralization and privatization of resource management in Africa (Büscher 2014; Fletcher, 2010: 172). This shift in conservation thinking has been widely criticized as a commodification of natural resources through wildlife tourism and as a neo-liberal marketisation of conservation (Bluwstein, 2018; Igoe and Brockington, 2007: 438; Büscher 2014; Ferguson, 2006). To participate in the model, landholders must comply to the market rationales of payments, accountabilities and linked technologies (Goldman, 2001; see also Büscher and Dressler, 2007: 596). The benefits of the land reforms which promoted the privatisation of pastoral land in Kenya are also highly debated. Apart from lack of transparency and injustices in the process itself (Bedelian, 2014; see below), they reduced mobility and access to water and dry resources (Kieti et al., 2020). In addition, landholdings have become increasingly fragmented into sizes which cannot support viable agricultural or livestock production (Pas Schrijver, 2019). This has increased dependency in tourism as an income and has in many areas of Kenya led to conflicts between pastoralists and conservation areas (Kieti et al., 2020). In the Maasai Mara region this debate is particularly sensitive due to the legacy of exclusion and land reallocation. The 20^th^ century conservation policy in Kenya arose from wilderness type national parks with eviction of communities or their exclusion from resource use (Cockerill and Hagerman, 2020; Igoe and Brockington, 2007: 43; Waithaka, 2012), a pattern common to other parts of Africa (Anderson and Groove, 1987; Brockington, 2002; Hutton, et al., 2005; Neumann, 1998). The legacy of conservation and land reforms has implications for how the effects of the Covid-19 pandemic was discussed in the MMWCA in terms of equity and benefit sharing. To understand this further we need to give a short historical context and background to the creation of the MMWCA.

## Context

The Maasai Mara National Reserve (MMNR) was established in 1961, under the Narok County Council (NCC), with highly restricted human activity. Prior to this, colonial land privatization had large scale effects on local communities, especially nomadic pastoralists such as the Maasai (Kabiri, 2010; Matheka, 2008). Although new policies and the establishment of the Kenya Wildlife Service (KWS) in 1989 were aimed at benefit sharing, equity, and increased community representation, these were riddled by corruption and problems of misrepresentation from local political elites (Waithaka, 2012).

In 1968, the Kenyan government also promoted the creation of group ranches, giving legal tenure to community groups with the ambition to commercialise Maasai livestock production (Bedelian, 2014; Thompson, et al., 2009). The previously open rangeland was divided into 11 smaller group ranches (Mwangi, 2007). A decade later these were dissolved into individual land titles – a process driven largely by local interests over concerns of inequity and corruption (Bedelian, 2014; Galaty, 1992; Kimani and Pickard, 1998; Mwangi, 2007: 890). By the 1990s, bilateral funding and investments made wildlife tourism lucrative and the number of private conservancies had increased in Maasai Mara (Cockerill and Hagerman, 2020; King, et al., 2016). In the early 2000s, several of the individual landowners came into agreements with private sector investors to form wildlife conservancies.

Today, there are 16 registered conservancies of varied size, with a total of 14,528 Maasai landowners (MMWCA, 2019), each with different solutions to benefit sharing (Bedelian, 2012; Broekhuis, et al., 2020; Figure 1). The interests of Maasai landowners are therefore of high priority, as it is seen as paramount to the success of the model (MMWCA, 2019; Weldemichel and Lein, 2019). However, the subscription of landowners to the conservancy model not only pushed members into a business and conservancy rationale as discussed above, it also excluded those with less optimal land (Mwangi, 2007). In 2015, after some political strife and negotiations within the community, a new institution, the Maasai Mara Wildlife Conservancies Association (MMWCA) was created, run by a council bringing together landowners, tourism partners, local communities, conservancy management, with representation from NGOs and government institutions. The division between members and non-members is a function of history, and not all members of the community are landowners (Osano et al., 2013). Meanwhile some landowners have not conscribed to the model, which carries a high risk of disenfranchisement for some households. Fencing of non-member property creates conflicts with wildlife and increases the burden of wildlife rangers employed by the conservancies (Broekhuis, et al., 2020; Nkedianye et al., 2009). The model also locks partners into non-use of areas, as grazing of livestock is only allowed inside some conservancies at specific times during off-peak season. Some conservancies do not allow grazing at all (Bedelian, 2012). When land-lease payments are too low or household cost increase, expanding grazing may be the only option for landowners who are now under binding contracts. As members instead tend to graze their cattle outside the protected areas it deepens the inequity for the non-members who are now forced to share pasture (Weldemichel and Lein, 2019). In addition, non-members are also affected by the impact of conservation in terms of wildlife damages, yet they do not reap the same benefits as members. Incomes emanating from conservation are also concentrated to few households (Homewood, et al., 2012; Osano et al., 2013). These inequities and conflicts were also highlighted during the Covid-19 pandemic as will be discussed here. (Table 1)

**Table 1.** Conservancy areas of the Kenya Maasai Mara Wildlife Conservancies Association (MMWCA) and their organisational structure.

**Enonkishu** (2011) 42 landown. And 2 fac.; partnership between Olerai Mara Farm management and surrounding landowners
**Isaaten** (2012) 318 landown., 1 fac.; Part of the greater Siana, formed through collaboration of Bushtop with landowners
**Lemek** (2009) 480 landown., 5 fac.; **Naboisho** (2010); 609 landown. 8 fac.; Amalgamation of 4 Lemek associations and the Koyaki-Lemek wildlife trust
**Mara North** (2009); 696 landown., 13 fac.; Amalgamation of the 12 associations managing the Koiyaki section of the Koyaki-Lemek wildlife trust
**Nashulai** (2016) 63 landown., 1 fac.; Partnership between local community member and landowner in the area adjacent to MMNR
**Motorologi-Olare Orok (now Motorogi)** (2006–7) 292 landown. 5 fac.; Fusion of Olare Orok formed by landowners w. support from tourism partners and Motorogi conservancy formed by landowners supported by Virgin
**Olarro** 2,200 landown., 2 fac.; Expanded 2016 to form North and South Olarro
**Olchorro Oiroua** (1992); 177 landown., 4 fac.; Formed by Olchoro Oiroua wildlife association
**Ol Kinyei** (2005) 240 landown., 4 fac.; Formed from Ol Kinyei group ranch by one investor, Porini camps
**Olderkesi** 2016; 7,000 landown., 4 fac.; Partnership between local community and tourism partner Cottars
**Oloisukut** (2010); 65 landown., 2 fac.; Formed from Kimintet group ranch, relaunched in partnership with WWF in 2016
**Pardamat** (2016) 850 landown., 0 fac.; created from Block 3 of the former Koiyaki group ranch
**Siana** (2016) 1,484 landown., 5 fac.; Formed from Siana group ranch in 2001, re-launched in partnership with WWF in 2016

## Research Methods and data collection

Qualitative semi-structured interviews (cf. Bernard, 2011) were carried out with 20 different stakeholders (Tables 2 and 3). Questions were formulated around the history of each conservancy and the negotiation of regulations, institutions and practices of the conservancy more broadly (Fletcher, 2017). In this paper we will focus specifically on responses to the Covid-19 pandemic in terms of effects and responses and the debates on equity and benefit sharing which ensued with decreased incomes.

**Table 2.** List of interviewees, their occupation, roles and organisation affiliation (date of interview and location).

Name	Occupation/Role	Organisation	Date	Location
Anonymous 1	camp employee/son of landowner	n/a	19/01/2021	Olare Motorogi conservancy
Andreas Fox	Tour operator	Ker and Downey safaris limited	10–02-2021	Zoom
Crystal Mogensen	CEO of MMWCA affiliate org	Maa trust	18/01/2021	Maa trust HQ, Olare Motorogi conservancy
Anonymous 2	MMWCA employee	MMWCA	19/01/2021	MMWCA HQ, Talek Town
Anonymous 3	Landowner/camp employee	n/a	22/01/2021	Ol Kinyei conservancy
Anonymous 4	Employee of MMWCA affiliate org	n/a	03/02/2021	Zoom
Isen	Employee of MMWCA affiliate org	Maa trust	01/03/2021	Zoom
			06/05/2021	Email
Jackson	Landowner/senior guide	Ol Kinyei conservancy	18/01/2021	Olare Motorogi conservancy
John	Landowner/camp employee	Ol Kinyei conservancy	21/02/2021	Ol Kinyei conservancy
Jui Banerjee	Camp owner	Porini Cheetah camp	03–04-2021	Nairobi
Kasmira Cockerill	Employee of MMWCA affiliate org	Maliasili	25/01/2021	Zoom
Micheal	Employee of MMWCA affiliate org	Koiyaki guiding school	21/01/2021	Naboisho conservancy
Mohanjeet	Tour operator	Gamewatcher’s safaris/Porini camps	27/01/2021	Nairobi
			17/04/2021	Whatsapp
Rebekah NN	Conservancy manager (non Maasai)	Enonkishu conservancy	25/02/2021; 2/4 2021	Zoom
Anonymous 5	Camp employee (non Maasai)	n/a	20/01/2021	Olare Motorogi conservancy
Simon	Landowner/Conservation warden	Ol Kinyei conservancy	20/01/2021	Ol Kinyei conservancy
Anonymous 6	Landowner/camp employee	n/a	20/01/2021	Ol Kinyei conservancy
Thomas	Camp employee (non Maasai)	Porini Mara camp	20/01/2021	Ol Kinyei conservancy
Anonymous 7	camp employee/son of landowner/conservancy youth leader	n/a	20/01/2021	Ol Kinyei conservancy
Anonymous 8	Landowner/camp employee	n/a	20/02/2021	Ol Kinyei conservancy

**Table 3.** List of interview questions, and the thematic analyses of responses.

Questions asked to the different stakeholders	Landowners	Hotel employees	Cons. Association staff	Wildlife rangers	Hotel/lodge owners	Tourism operators	Ministry/tourism board	Related themes
Tell me about yourself and how you ended up here, explain your role in the organisation?	x	x	x	x	x	x	x	—
The conservancy model								
How does the conservancy model work, what are the benefits challenges, improvements for yourself or community?	x	x	x	x	x	x	x	3, 5,6
Tell me how communication between stakeholders works, describe the internal relationships and roles?	x	x	x	x	x	x	x	2,3,5, 6
Why did you choose to become a member; what other incomes do you have; Have you ever thought about leaving the conservancy model, what would you do instead?	x	—	—	—	—	—	—	2,3, 5,6
Why did you choose to have your lodge in a conservancy rather than inside the Park? What type of agreements do you have with landholders?	—	—	—	—	x	x	—	2,3, 5,6
How does working in the conservancy differ from working in the reserve; what are the specific challenges; do you compliment incomes with other revenues?	—	—	x	x	—	x	—	3,5, 6
Organisational values/strategies								
What does sustainability mean to you or your organization? How do you try to incorporate this into your work?	—	x	x	x	x	x	x	1,5,6
How do see the link between climate change risks and global tourism, does tourism need to change?	—	—	—	—	x	x	x	1,4, 5,6
What were your strategies to bring up tourism numbers how were they different to the kinds of strategies being used right now	—	—	x	x	x	x	X	4,6
Covid-19								
Can you think of any other times when tourism was impacted in this way (i.e. financial crises, terrorism attacks, other events), what were the similarities/differences?	x	x	x	x	x	x	x	1,5, 6
How did Covid-19 affect you this year?	x	x	x	x	x	x	x	1,2
What measures did you to mitigate negative effects and when restrictions were opened up again?	—	x	x	x	x	—	x	2,3, 4,5
Did you get any external assistance, how did it work?	x	—	x	x	x	—	—	2,3,5
Do you think the Mara and other conservancies will now rely more on local tourism? Why or why not?	—	—	x	x	x	x	x	4,5, 6
Future								
Do you think that the slow-down of tourism will impact the conservancy model, how and why? Do you think that Covid- 19 pandemic will have long term impacts on tourism industry	x	—	x	x	x	x	x	1,6
If it doesn’t pick back up would you look for work in a different industry/change livelihood and contracts?	x	x	—	—	x	x	—	4,5, 6
Themes								
Theme 1: How does the Covid-19 pandemic compare to other crises								
Theme 2: Securing lease payments (steps taken and external support)								
Theme 3: Effects on local economy and mitigation strategies								
Theme 4: The influence of domestic tourism (and it’s future potential)								
Theme 5: Equity and benefit sharing (in light of the negative effects on economy during the pandemic								
Theme 6: The future of the conservancy model and tourism in general								

Interviewees were selected to represent a diversity of partners while also having some insight as representatives of an organisation or interest group within MMWCAParticipants were also selected through by recommendation from other interviewees (so called snowballing, cf Bernard, 2011). Of the interviewees, 10 were staff in the conservancies, six of which were landowners, and two were members of a family of a landowner. Other interviewees were tourism operators and staff members from the Maa Trust, as well as employees of MMWCA and Kenya Wildlife Trust (Table 2). All interviewees were informed of the purpose of the study and signed a consent form or agreed verbally to consent. They were informed of their rights to confidentiality and the option to withdraw at any time. The interviewees who preferred to appear as anonymous are here quoted as anonymous and organisation affiliations have been redacted. Most interviewees preferred to appear with their first or full names and with their organisation named. Interviews were carried out in January 2021 in the Maasai Mara, typically in the workplace of the interviewee (Table 2). Several interviews were conducted online or complimented in the subsequent months via zoom.

The interviews took between 45 min to 1 hour. Interviews were recorded and then transcribed. Here we have selected those questions relating to the conservancy model, organisational values strategies, the direct effects of the Covid-19 pandemic and steps taken, and the future which directly or indirectly came to be influenced by the Covid 19-pandemic, (Table 3). Questions relating to the conservancy model and organisational values also came to centre around the ongoing pandemic which is why these questions are also shown here. Notes were taken on the patterns and themes that emerged from the transcription. The whole body of transcripts and field notes were coded and thematised in an excel spreadsheet building out interrelationships between the themes (Minichiello, et al., 2008: 5; Oltmann 2016). All interviewees were given the opportunity to read and comment on the transcripts. Interviewees were sent the drafted paper for comment in February 2022 and were asked for consent to participate in this paper (cf. OHO 2022), which also allowed us to follow up on some information.

## Findings

Below we present the results of the interviews based on the six themes which directly or indirectly related to the Covid-19 pandemic (Table 3). The first related to how the effects of the Covid-19 related to previous shocks. All interviewees agreed that the Covid-19 pandemic had effects on a scale which had never been experienced before (as compared to past effects from terror attacks, recessions in the global economy, etc.). Another difference according to the interviewees has been the level of uncertainty around the spread of the pandemic and changing regulations. The other themes related to how to 2) secure funding, 3) the impact on local economies and solutions taken, 4) the influence of domestic tourism. The results of the interviews relating to these themes will be presented below focusing on the MMWCA.

### Securing funding in the conservancies

During the early phase of the pandemic, March-April 2020, MMWCA provided overarching support for all its members to secure land lease payments and keep conservancies in operation until tourism recovered. It was quickly realised that without any tourists even tourism operators with long established businesses and relationships with the Maasai community were unlikely to be able to fulfil their commitments beyond the first few months. Lack of funds also affected the conservancies’ operational costs, including ranger’s salaries. However, because each conservancy acts as an individual legal entity, they approached the pandemic in different ways.

#### Securement of funds

A Crisis Committee was established promptly in March 2020 by the MMWCA consisting of its board members, tourism partners and friends of the association. The first task of the Crisis Committee was to ensure that the community had access to the conservancies for their pastoralist activities, as this is a significant source of income aside from the lease payments. A representative from the Msaloasili organisation, who is a partner of MMWCA and who worked with the committee, explained how these efforts entailed direct fundraising for philanthropic and donor finance (Kasmira Cockerill 25/01/2021). In addition, MMWCA was involved in the coordination and support of fundraising for an “Operating Expenses” fund which, according to an anonymous member of the Crisis Committee, supported 12 out of 16 conservancies on their monthly operational budgets. This funding was also supported by the 1 billion Kenya shillings ($9300000, based on the exchange rate of 31/04/2021) dedicated to private wildlife conservancies from the government stimulus package in June 2020 (KWCA, 2020).

The main source market for the conservancies are the UK and USA, which were among the countries worst-affected by Covid-19. Conservancy camps work on the basis of forward bookings, many of which were postponed and some cancelled, severely affecting the tourism partners’ cash flows. An anonymous informant who works with MMWCA explained that two tourism partners were forced to back out of their agreements with their respective conservancies because they were not able to stay afloat under the conditions of the pandemic, and therefore were unable to pay their obligations to the landowners.

Employment was also affected. In one conservancy, employees agreed with the management to receive 30% of their salaries during the initial lockdown. After the removal of the inter-county lockdown in Kenya in August 2020, salaries were increased to 50% as domestic tourism picked up slightly. Similar arrangements were made in other conservancies, though some still had to lay off employees. Other systems included rotational shifts whereby employees worked for 1 month and then went on unpaid leave for another month, in order to reduce operational costs without dismissing staff members.

#### Renegotiation of leases

An important goal for the Crisis Committee was to ensure the continuity of the leases. Some lease contracts had as recently as 2019 been renewed for the coming 25 years, but the pandemic risked enforcing a force majeure clause. Therefore, MMWCA mobilised a communications campaign with the landowners which aimed to:

*[…] renegotiate the leases and create revenue streams of options around that so that the conservancy as an institution, as a collection of individual landowners and the leases themselves remain viable through this* (Kasmira Cockerill 26/01/21)*.*

The MMWCA and partners agreed to reduce lease payments to 50% across all conservancies until June 2021, after which outstanding lease payments would be repaid if tourism occupancies reached a certain level (the sum was not disclosed in the interviews). The Crisis Committee had the ambition to raise additional funding to support the tourism operators to pay the remaining 50%. Therefore, MMWCA entered a partnership with Conservation International Ventures (CIV), which provided soft loans to tourism partners with repayment contingent on the recovery of the industry.

#### External support

The crisis funding provided by MMWCA provided some support to conservancies, but it was not enough. Especially important was the network of small private family foundations in the USA. The payment of the land leases was, and still remains, the main priority across all conservancies because it is the foundation of the entire model. The Ol Kinyei Conservancy is a good example of the complexity of payments. The conservancy is managed by Porini Camps and covers 18,700 acres and has 81 employees from the community. According to the Managing Director of Porini Camps, it costs $639,950 to pay the leases on that land and to keep the community employed each year, and the annual cost of leases and salaries is $35 per acre per year. To ensure continued lease payments, Porini Camps, as the sole tourist partner at Ol Kinyei Conservancy, organised a fundraising campaign targeted towards repeat clients who were encouraged to ‘adopt’ an acre to the calculated sum of $35. Individuals who adopted more than 30 acres, amounting to $1050, received a credit on future travel. The Managing Director elaborated on this initiative:

*There are people out there that want to do good but they would also like to get something in return so that is actually a way they can make a difference to us now, and at the same time, have it as a credit towards a future safari*.

As of January 2021, Porini Camps was able to raise $280,000 through this program.70% came from people who had adopted 30 or more acres, and as a side effect, future clients for the conservancy were secured. However, the funds are not limited to Porini Camps’ facilities in the Mara, as they have camps in conservancies all over Kenya.

By contrast the Enonkishu Conservancy operates as a mixed-use conservancy with communal cattle herds, one tourist facility and part of the acreage owned by private individuals that fall under the Naretoi Homeowners Association. The homeowners pay the conservancy a fixed fee every 6 months, regardless of occupancy, and this has eased the conservancy’s burden to fulfil land lease obligations through the pandemic. Furthermore, homeowners rented out their properties on platforms such as Airbnb and, when the inter-county lockdown was lifted in July 2020, many domestic tourists rented these accommodations. One official from the conservancy described this process to us.

Tourists paid daily conservancy fees in addition to that paid by the homeowners, providing extra income for the conservancy to run its operations. In addition, Enonkishu received funding from an organisation called For Rangers Kenya, which ensured that rangers remained in employment and could protect the wildlife, livestock and people in the conservancy and its environs. Before this, Enonkishu was in a similar position to other conservancies where they had to cut wages in half and reduce costs where possible, as explained by an official from the conservancy: “*for a month or two, we were, you know, really minimising how much fuel we used and not fixing the cars or the motorbikes, and basically everything was grounded*”. However, other conservancies were not as fortunate to receive additional funding, and some were relying completely on MMWCA to run their operations.

A positive impact seen during the pandemic was the outpouring of donations from unexpected people, according to one anonymous conservancy manager:

…*prior to Covid we really struggled to find donors that could significantly support projects […] we [would get] a cheque for $50, but during Covid it was like, we got a cheque for $10,000, people really stepped it up*.

The conservancy proactively increased its online presence and community engagement on social media during the pandemic, and the manager saw a direct correlation between media presence and donations. With people all around the world being confined to their homes, internet and social media usage surged and many organisations used this as an opportunity to increase their networks. Another conservancy manager explained how they had used a similar tactic – they had shared “virtual safaris” on their social media pages in an attempt to keep their online community engaged.

### Effects on local economy

The entire Mara region depends on tourism in one way or another for their income. Covid-19 therefore had a huge negative impact on the region. A particular example of this was explained by a landowner who is employed as a camp manager at a conservancy:

…a trading centre like Ololulung’a [which] is just by the road to the main entrance of MMNR... it is a stop-over centre where a lot of tourists stop by the way, having some sodas and drinks and all that. So now you don’t see that flow of money, no people, no customers.

A number of small shops, petrol stations, car washes, restaurants and curio markets are located along the way to the conservancies. While these are not official statistics, it is estimated that hawkers, kiosks and small businesses can earn up to $50 each day from tourists (Reuters, 2020).

#### Effects on community

Non-members, lacking stable incomes, were severely affected by the pandemic, as businesses and cattle markets closed down. The decrease in landowners’ payments also affected the whole community, as one anonymous camp employee described it:

…*it’s like an inter beneficial [relationship] because when the company pays the landowners, the landowners also share what they get to the non-members so that actually they can also get their basic needs*.

The most severe challenge during the pandemic, according to the same informant, was food shortages due to lack of income.

Tourism partners at the beginning of 2020, pre-pandemic, were investing in a number of community projects, as 2019 had been a successful year in terms of tourist numbers and revenues. However, the onset of the pandemic meant that lease payments became the main priority, and all other projects were postponed. Several interviewees described the community’s disappointment when projects had to be put on hold. For instance, Porini Campswas forced to postpone its planned development projects, as explained by an employee:

*We had a school [where] we were to build a classroom because they were short of classrooms… We [had] actually looked for the money and the time, the business was getting good and then all of a sudden there was all this problem*

Another example of cascading effects is the Maa Trust, a non-profit that works with the community to implement development projects and promote entrepreneurship. The Trust has several ongoing programs, including in areas such as WASH (eg. Child Rights, Water, Sanitation and Hygiene-Healthcare), which are now underfunded. This is also partly due to the fact that fundraising takes place mainly in the UK through charity auctions and balls which also were halted. Their biggest project is Maa Beadwork, in which local women craft jewellery and other items to sell to tourists. The Beadwork Project provides income for over 500 Maasai women from both conservancy and non-conservancy member families. According to the Trust’s CEO, Dr Crystal Mogensen:

…*beadwork's main market is either selling to the camps, if they have a shop in the camp, or the camp sending their guests to our shop here. So that accounts for about 60-70% of our sales [referring to Maa Beadwork] each year, and that was immediately gone*.

This income is important for women who do not otherwise directly receive payments, and who are underrepresented as employees in the conservancies and linked hospitality services.

While the pandemic put a halt on some projects, the continued strength of the relationship between the community and the conservancy management, in this case Ol Kinyei, can be seen in the way that both parties handled the impact of the pandemic and negotiated to find a middle ground. A Porini camp manager explained:

*We talked to the community, that this is a global issue, it has affected the company, it has affected business, there is no tourists so now they accepted on a half lease payment because they know the business pays to the leases […] and that's why people have not come to demand their land so that has worked and that's why we are still maintaining our conservancy up to this level. And we're still talking to the community, that once things get normal, then everything will be paid normally, will normalise*.

Covid-19 impacted the national and regional economy severely, such that locals were feeling its impacts in their lives through the closure of schools and markets, etc. and were therefore understanding of the deductions and postponements that needed to be implemented.

#### Income diversification

Several interviewees stressed that this pandemic has opened their eyes to the need to diversify their sources of income. Camp employees, when discussing their experiences over the past year also described their time off during the initial lock down as an advantage. Before the pandemic, because of the remote locations of the camps, the work schedule was 6 weeks on duty and then 2 weeks off. One camp employee explained that this rotation made it challenging to balance other responsibilities, and normally he especially missed spending time with his family. Another tour guide explained how the crisis resulted in better care of his cattle:

*When Covid came, everyone was at home so we were busy treating our animals and managing it well because [those who] are always [at] work will have to hire a shepherd [and] you know, the owner of the property will have to look [after] it properly, like we did well and then we found like oh, my cattle were not well treated when I was at work*.

Other Maasai interviewees described how they now had the opportunity to participate in the traditional ceremonies they miss out on when they are working at the camps. For example, a mess tent worker at one of the camps mentioned that he was able to partake in the traditional initiation ceremony (e.g. the *Enkangoo-nikiri*, the so-called meat camper ceremony), which he had not done for a few years because of his work schedule.

Some landowners engaged in various alternative income generating activities. One guide explained how people in his village had been expanding cattle rearing:

*When they are getting this month off and going for unpaid leave, some maybe go and start another business [...] you might start a shop or you might start like a business of taking cows to the big market like Nairobi Dagoretti, and you go to sell there and you get profit*.

This kind of creative negotiation within the confines of the pandemic was a common anecdote from many interviewees. Another conservancy employee mentioned how many people had started selling milk to national dairy companies. The heavy rains in 2020 aided in this process:

[…] *we were blessed by that time because the rain was [there] throughout during the Covid time and our animals, our cattle [had] plenty[of] food so they produce so much milk”*.

He went on to explain that his wife earned 30,000 kenya shillings ($279) in a month from selling milk. This income is high, considering that the highest sum of a lease payment for a large parcel of land in a core conservancy amounted to 26,000 Kenya shillings ($242) (based on information obtained from the interviews).

### Domestic tourism

Domestic tourism to conservation areas is typically low in Kenya, and prices in the conservancies are typically set high. There is a wealthy and discerning market in Kenya that was willing to pay the high international rates set by some camps, especially as international tourism was not possible. When the inter-county lockdown was lifted in August 2020, domestic tourism increased. Some informants described how domestic tourists were “delighted” with their visits to the conservancies, and that they are likely to come back.

While the upsurge of domestic tourism provided much-needed income to the conservancies, most of the stakeholders interviewed do not believe that reliance on the domestic market is financially viable. One reason frequently stated by the interviewees is the disparity between the conservancy fees for local and international tourists. Therefore, at least in the eyes of one tour operator the fees generated by local tourists are not considered sufficient to maintain conservancy operations:

*The conservancies […] because some of the fixed costs are so high, because you've got very small bed numbers and you've got high lease fees, they're struggling to compete on rates* (Andreas Fox Zoom 10–02–2021)

Citizens and residents of Kenya pay a significantly lower daily rate than international tourists; 2000 Kenya shillings ($18) in comparison to $85 for international tourists. Accommodation is also significantly more expensive for foreigners than for locals. Another challenge expressed by hospitality and other tourism partners is that Kenyan tourists tend to travel primarily during holiday seasons and over weekends. As explained by one informant: *“Even if you’re full four nights a month or eight nights a month, you can’t run a business like that, you can’t cover your costs.”* These short bursts of visitation are not feasible because they do not significantly contribute to conservancy operation costs or business costs.

## Discussion

The effects of Covid-19 on decline in international tourism has been higher than eve measured before in any other crises (Duro et al., 2021; Gössling et al., 2020; UNWTO, 2022), a fact which was confirmed by all interviewees. In addition, tourism operators around the world were faced with reduced revenues and increased costs as they implemented protective Covid-19 measures (Fotiadis et al., 2021), and this was also the case in Kenya. Camps and lodges in the Masai Mara conservancies had to follow Ministry of Health protocols, with significant costs. The MMWCA proactively intervened at an early stage, developing a clear proposal of needs, and then lobbied donors fitting their needs. A representative from the Crisis Committee, stressed how this approach is otherwise uncommon because *“usually the broken system is the other way around, with organisations bending over backwards to meet donor interests”* (Kasmira Cockerill 25/01/2021). Landowner’s acceptance of the 50% deduction in lease payments was based on MMWCA’s campaigning. One conservancy manager mentioned that the small size of their conservancy was an advantage because they had personal relationships with all the landowners which made it easier to convince them to agree to MMWCA’s terms. The re-negotiations of the leases were no small feat, since the MMWCA had to coordinate with close to 15,000 landowners at a time when physical meetings were prohibited. However, its longer-term negative effects in terms of trust towards the payment system must now be followed up closely.

Tourism numbers were a record high in 2019 and typically a 35–40% occupancy is enough to meet financial obligations in terms of lease payments and salaries (MMWCA, 2019). Still, when the pandemic began in early 2020, conservancies could not pay landowners. The forum created by MMWCA to negotiate the temporary payment reduction therefore became a space to debate the lack of equity as the process itself provided a tangible example of the uneven distribution of profits from the model. Already before the pandemic, and now strengthened by these debates, alternative structures to the lease payments have been proposed by several stakeholders, which seek to incorporate the landowners more thoroughly by increasing transparency from the tourism partners. Several interviewees also brought up the rights of women and the place of Maasai youth in the future of the model. It will be interesting to see how these ideas are negotiated by the stakeholders in the months and years to come.

One landowner described the pandemic as “*a blessing and a curse*”. While Covid-19 of course had significant impacts on the livelihoods of different actors in different roles, it created the space for people to reflect on their lives. As shown above, some Maasai camp employees enjoyed the time away from work because they got to spend more time with their families, and because they could tend to their livestock. Others experimented with alternative income sources and one landowner commented that the guaranteed payments from their land-leases had made him complacent, but the pandemic forced him to be more proactive. The time away from the conservancies opened many landowner’s eyes to the possibilities for alternative land use, and gave them pause to think about whether conservation is their best option both in terms of economy and time. Although some informants described a sense of reverence for the wildlife, most described it as a tool for income generation (see also Sachedina and Nelson, 2010; Osano, et al., 2013; Broekhuis, et al., 2020). The diversification of incomes during the pandemic is positive from the perspective of the Maasai, but it could prove to be problematic for the future of the conservancy model. To increase possibilities of income diversification there needs to be more openness towards mixed land-use leases. Pardamat Conservation Area (PCA), a newer conservancy, is an example of what can be achieved by promoting mixed land use practices. It is the only conservancy in the Mara landscape with the premise of a dual-use conservation model. 850 Maasai landowners have legally registered their 26,000 acres as a wildlife conservation area, while continuing to live and work in the area (MMWCA, 2019). We were not able to speak with stakeholders from this conservancy, but it will be interesting to see how the mixed land-use model develops into the future.

Domestic tourism in some ways, after national restrictions were lifted, did compensate for losses and also possibly opened up a new market. However, the representatives of hospitality services do not at the moment see domestic tourism as a long-term option. Domestic tourists generate as low as a quarter or a third of the revenue that international tourists do, but the cost of operating the camps remains the same. Conservancy tourism facilities maintain few beds per camp and are reluctant to expand their accommodations due to the negative environmental impacts of higher visitor numbers. Therefore, camps typically cannot afford to reduce the cost per bed night. Since dometic tourists are not able to same pay the same prices as international tourism, prices impact their level of visitation to the conservancies. Elsewhere in Africa price levels have been shown to be strong deterrents for domestic tourism (Melubo, 2020; Woyo, 2021). The Covid-19 pandemic has reminded us of the importance of a domestic market during global crises (Rogerson and Baum, 2020; Soliku et al., 2021; Hambira et al., 2022) and its potential when it comes to income redistribution and poverty mitigation (cf. Llorca-Rodríguez et al., 2020). More efforts should therefore be made to encourage domestic tourism, which is also important for the anchoring of conservation efforts nationally.

Post-pandemic, many informants expect travel to change, both in the ways that tourists choose their destinations and in the way that destinations operate. The pandemic generally allows us to reflect over international tourism, its negative effects in terms of emissions and the problems of benefit sharing and equity (Hall et al., 2020; Niewiadomski, 2020; Gössling and Higham, 2020). The conservancies provide a more exclusive and expensive experience which, many informants believed, will be an advantage in the wake of the pandemic as the high-end target market is relatively economically stable. Tour operator, Andreas Fox, suggested that *“tourism across the world will become slightly more focused on quality over quantity”.* This would be reflected in the development of more discerning tourists who would be more selective in choosing the destination, from a perspective of safety but also in terms of the value of the experience. Still, many interviewees, especially those from hospitality management, stressed that MMWCA needs to develop diversified tourism products within the region in order to increase visitation overall and to secure multiple sources of revenue.

MMWCA has taken lessons from Covid-19 to develop long-term strategies to mitigate the impacts of any potential future shocks. One idea is the establishment of a reserve fund to act as a buffer that would support conservancies in times of conflict or crisis. This is especially important in a model like Maasai Mara where conservation must take place in the broader landscape and with many partners operating differently. This kind of reserve fund would require 8–10 years to mature and, according to an MMWCA employee, will not be a priority for the organisation until 2022 at the earliest. Another long-term plan is the development of a marketing strategy encompassing the entire region into a ‘One Mara’ brand. The One Mara brand could potentially bring together tourism partners, the Maasai community and other stakeholders to develop a distinctive brand in order to increase exposure and eventually attract more tourists.

## Conclusion and recommendations

The Maasai Mara Wildlife Conservancies Association (MMWCA) is an amalgamation of stakeholders who vary greatly in many aspects. Potentially the model has multiple benefits over traditional park models as landholders are formal partners in the conservation model (see similar discussion in Hulme and Murphree, 2001; Nelson et al., 2010; Sachedina and Nelson, 2010; Davis and Goldman, 2017). Community owned conservancies have also been shown to perform well in terms of wildlife densities (Western, et al., 2009, 2020). However, as discussed in the introduction, nature-based and wildlife tourism are generally dependent on international tourism and sensitive to global trends. As we have seen, the sensitivity is particularly high where community payment is funded through revenue (Hockings et al., 2020; Lindsey et al., 2020; Mudzengi et al., 2022). Therefore, it is of interest to assess the effects of the Covid-19 pandemic and its mitigations as done here.

MMWCA’s swift action enabled them to find more flexible and dynamic sources of funding. In addition, the re-negotiations of the leases with landowners was an achievement in terms of collaboration and trust. A major development – arguably the most significant – through the pandemic has been the solidification of MMWCA’s position of authority in creating a shared vision, aims and goals (cf. Beeton, 2006: 58–60). The importance of partnerships for the mitigation of negative Covid effects were also strongly stressed by the World Travel and Tourism Council report on the pandemic (WTTC, 2021). In the case of MMWCA, the organisation’s quick and proactive response was crucial with the formation of the Crises Committee. Strategies from the Crisis Committee included fundraising, communication with the landowners and its overall management of the pandemic increased its power by tangibly demonstrating its value as a coordinating body. In addition, conservancies by increasing activity on social media, were able to attract donor funding and also to draw tourists post-pandemic through marketing and donor campaigns. The government stimulus package also did have decisive positive effects, which shows the importance of governmental support to the tourism industry (see also WTTC, 2021 for a similar discussion).

The pandemic has shown operators the potential benefit of also targeting domestic tourists which also has the potential to better spread the distribution of revenue. The current reliance on international tourism is a general problem faced by conservancies in Africa and low-income countries elsewhere where financing is based on revenue rather than tax funding. At the moment operators do not see that domestic tourism could supplant revenues from international tourism, but it is clear from the pandemic that it is definitely important as a compliment to international tourism. A recommendation is to introduce additional compensatory payments for domestic tourism to allow a low price but price compensation for operators – this would be important to anchor conservation more broadly among the Kenyan public. The Covid-19 pandemic has highlighted the need for identifying alternative sources of revenue for the MMWCA as a whole. These lessons are important not just for MMWCA but also for the future of other conservancies in Africa.

As a result of the pandemic, land lease payments were temporarily reduced to 50% and many workers in conservancies and camps were either put on a rotational work scheme or given longer times on leave, while some were also laid off. The crisis also affected the local economy overall. Generally, the need for income diversification was stressed by landowners. Some landowners were able to transition to dairy production or to intensify cattle rearing. The potential revenues from such activities, and the realisation that lease payments are not as stable as assumed prior to the pandemic, may be a risk to the model itself. The effects of the pandemic emphasized deep divides in income, resources and social standing which must be addressed over the coming years. Clearly, if lease payments are too low or unstable, landowners will be more reluctant to participate in the model. The pandemic also stressed the benefits of land lease agreements and arrangements that encourage adaptable land-use that can respond better to crises and it is recommended to develop systems for multiple paths of livelihood and land-use in the future.
